# Root Exudates from Coexisting Plant Species Differentially Shape Soil Microbial Communities and Nutrient Dynamics in a Desert Steppe

**DOI:** 10.3390/microorganisms14050950

**Published:** 2026-04-23

**Authors:** Leqing E, Guodong Han, Jie Liu, Xuefeng Gao

**Affiliations:** 1 College of Life Science and Technology, Inner Mongolia Normal University, Hohhot 010022, China; elqin0117@163.com (L.E.); lj18175071216@163.com (J.L.); 2 College of Grassland Science, Inner Mongolia Agricultural University, Hohhot 010022, China; nmghanguodong@163.com

**Keywords:** desert steppe, root exudates, soil microbial community, nutrient properties

## Abstract

Root exudates are key drivers of rhizosphere microbial assembly, yet their effects across coexisting plant species with different functional roles remain unclear. We examined the effects of root exudates from five desert steppe species in Inner Mongolia: one constructive species, two dominant species, and two accompanying species. Exudates were collected hydroponically and applied to bulk soil in a three-week incubation experiment. Microbial communities were analyzed using high-throughput sequencing, functional prediction, and co-occurrence network analysis. Exudate addition significantly altered bacterial community composition, reducing bacterial richness, while fungal communities showed weaker responses. Exudates from constructive and dominant species enriched Actinobacteria, including *Rubrobacter*, *Arthrobacter*, and *Solirubrobacter*, and increased functional groups linked to chemoheterotrophy and nitrogen transformation. In contrast, exudates from accompanying species induced distinct microbial assemblages without promoting Actinobacteria dominance. Exudate addition also increased bacterial network complexity, suggesting enhanced microbial interactions. Soil pH decreased and available nitrogen and phosphorus increased, strongly correlating with bacterial community shifts. Overall, root exudates mediate species-specific microbial assembly and functional reorganization in desert steppe soils, driven mainly by plant functional roles rather than taxonomic relatedness. This study provides new insights into how plant-derived substrates regulate microbial communities and nutrient cycling in arid ecosystems.

## 1. Introduction

Root exudates are key mediators of material exchange and information transfer between plants and rhizosphere soils, and play an important role in shaping rhizosphere microenvironments [1,2]. Previous studies have shown that root exudates play critical roles in regulating soil biogeochemical cycles, rhizosphere ecological processes, and plant growth and development [3,4,5]. In particular, root exudates are widely recognized as key drivers shaping the composition and functioning of rhizosphere microbial communities [6,7]. As complex mixtures of organic compounds, root exudates provide carbon and energy sources for soil microorganisms [8,9], and variation in their composition and quantity among plant species influences microbial community composition, abundance, and activity [10,11]. Consequently, different plant species often support distinct rhizosphere microbial assemblages through differences in root exudate release [12,13].

Grassland ecosystems are characterized by high plant diversity and complex ecological interactions. During long-term adaptation to environmental conditions and natural succession, plant individuals compete for both aboveground and belowground resources while simultaneously influencing the growth and development of neighboring plants through root-derived metabolites [14,15]. These positive and negative interactions among plants are important factors underlying the spatial dominance patterns observed in plant populations. In the *Stipa breviflora* desert steppe in Inner Mongolia, plant communities develop through ongoing interactions, mutual adaptation, and selective processes among individuals competing for limited resources, resulting in zonal vegetation adapted to local environmental conditions. Vegetation in this ecosystem is characterized by a low and sparse grass layer and relatively low species richness, typically comprising more than 20 plant species. *Stipa breviflora* Griseb. is the constructive species, with *Artemisia frigida* Willd. and *Cleistogenes songorica* (Roshev.) Ohwi as dominant species, while *Convolvulus ammannii* Desr. and *Heteropappus altaicus* (Willd.) Novopokrov are common accompanying species [16,17]. Here, we examine whether root exudates from different plant species exhibit divergent ecological functions during interactions among coexisting species in desert steppe communities, and whether these differences are linked to interspecific competition.

Many plant species in the Inner Mongolian desert steppe share similar growth forms, with dominant species commonly exhibiting tussock or creeping growth habits, whereas tap-rooted species rarely become dominant [18]. For example, *S. breviflora* shows dense and progressively shallower root distributions as an adaptive response to grassland degradation, while *A. frigida*, a tap-rooted species, adapts to harsh conditions through clonal reproduction, shallow adventitious roots, and increased root branching [19]. Despite these observations, physiological and ecological adaptations related to survival and competition among these species in desert environments remain insufficiently explored.

Here, we examined the effects of root exudates on soil microbial communities and soil nutrient properties from five coexisting plant species in a desert steppe ecosystem of Inner Mongolia. Root exudates were collected using a hydroponic method, concentrated, and applied to in situ desert steppe soils in a three-week incubation experiment. Soil microbial community composition and diversity were assessed using high-throughput sequencing, together with measurements of major soil nutrient properties. We hypothesized that: (1) root exudates contribute to differences between rhizosphere and bulk soil microbial communities; (2) root exudates from plant species occupying different ecological roles exert distinct effects on soil microbial communities; and (3) functional differences among root exudates are associated with plant competitive ability within the community.

## 2. Materials and Methods

### 2.1. Study Area

**Figure 1:**
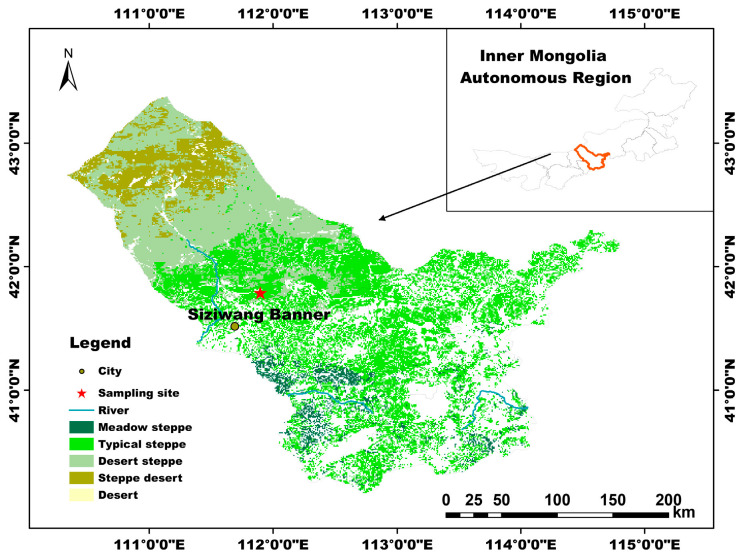
Location of the study site in the *Stipa breviflora* desert steppe of Inner Mongolia, China.

The experiment was carried out at the Siziwang Banner Experimental Station of the Inner Mongolia Academy of Agricultural Sciences, China (41°47′17″ N, 111°53′46″ E), at an elevation of 1456 m (Figure 1). The region has a typical temperate continental climate, characterized by dry and windy springs, short and hot summers, cool and rainy autumns, and long, cold winters, with pronounced seasonal variation. The mean annual temperature is 3.6 °C, and the mean annual precipitation is approximately 234 mm, most of which occurs between April and September.

The zonal vegetation is classified as *S. breviflora* desert steppe, with the grassland type dominated by *S. breviflora*, *A. frigida*, and *C. songorica*. Vegetation is sparse and low, with a community height of 8–10 cm and a total cover of 17–20%. Species richness is relatively low, with the community comprising more than 20 plant species. The soil is classified as light chestnut calcareous soil, characterized by low nitrogen and phosphorus availability, relatively high potassium content, low organic matter content, and a soil pH ranging from 7.80 to 7.99.

### 2.2. Root Sampling

Root sampling was conducted in August 2018 during the peak growing season in a grazing-excluded area of the *S. breviflora* desert steppe that had been fenced for 14 consecutive years. Fifteen individuals (or tussocks) were collected for each of five representative plant species, including the constructive species *S. breviflora*, dominant species *A. frigida* and *C. songorica*, and accompanying species *C. ammannii* and *H. altaicus*. Plants were excavated with root systems kept as intact as possible and transported to the laboratory, where roots were carefully cleaned using a soft brush for subsequent root exudate collection.

In addition, approximately 1 kg of bulk soil was collected from unvegetated areas of the grassland. Soil samples were air-dried, sieved through a 1 mm mesh, and used for incubation experiments involving the addition of root exudates.

### 2.3. Collection of Plant Root Exudates and Soil Treatment

Cleaned plants were first transferred to cultivation containers containing aerated Hoagland nutrient solution, with roots fully immersed, and allowed to recover for five days. After recovery, roots were surface-sterilized with 1% (*v*/*v*) NaClO for 20 min and thoroughly rinsed with distilled water. For each plant species, the 15 individuals were divided into three groups and individually placed into beakers using sponge supports. Sterile distilled water was added until the roots were completely submerged [20].

The beakers were wrapped with black cloth to exclude light and incubated at room temperature (28–30 °C) for 12 h. The resulting solution was collected as the 12 h root exudate sample. After filtration, the exudate solutions were concentrated fivefold under reduced pressure using a rotary evaporator (HB-10, IKA, Staufen, Germany).

Concentrated root exudates (5 mL) from *S. breviflora* (YD), *A. frigida* (YL), *C. songorica* (YY), *C. ammannii* (YX), and *H. altaicus* (YG) were each added to 50 g of air-dried grassland soil. For the control treatment (CK), 5 mL of sterile distilled water was added. The experiment included six treatments, each with three replicates. All soil samples were incubated at 25 °C for three weeks under controlled conditions.

### 2.4. Soil Microbial Analysis

The total genomic DNA was extracted from soil samples using the E.Z.N.A.™ Mag-Bind Soil DNA Kit (OMEGA Bio-tek, Norcross, GA, USA), and its integrity was confirmed through agarose gel electrophoresis. DNA concentration was determined using the Qubit 2.0 Fluorometer and DNA Quantification Kit (Thermo Fisher Scientific, Waltham, MA, USA) to ensure accurate DNA input for subsequent PCR reactions.

For the first round of PCR amplification, bacterial 16S rDNA was amplified using primers 341F (5′-CCCTACACGAGCTGCTTCCGATCTG-3′) and 805R (5′-GACTGGAGTTCCTTGGCCACCGAGAATTCCA-3′) to target the V3-V4 hypervariable regions [21]. Fungal ITS regions were amplified with primers ITS1 (5′-CCCTACACGAGCTGCTTCCGATCTN-3′) and ITS2-Rev (5′-GTGACTGGAGTTCCTTGGGCACCGAAGAATTCCA-3′) [22]. Each sample was barcoded to distinguish between sample origins. The PCR reaction mix (50 µL) included 10 × PCR buffer, dNTPs, genomic DNA (10 ng), primers, and Plantium Taq DNA polymerase. The PCR conditions involved an initial denaturation at 94 °C for 3 min, followed by 5 cycles of denaturation at 94 °C for 30 s, annealing at 45 °C for 20 s, and extension at 72 °C for 30 s; then 20 cycles of denaturation at 94 °C for 20 s, annealing at 55 °C for 20 s, and extension at 72 °C for 30 s, with a final extension at 72 °C for 5 min.

The second round of PCR amplified products with Illumina bridge-PCR compatible primers in a 50 µL reaction mix, with conditions involving denaturation at 95 °C for 30 s, followed by 5 cycles of denaturation at 95 °C for 15 s, annealing at 55 °C for 15 s, and extension at 72 °C for 30 s, and a final extension at 72 °C for 5 min.

PCR products were confirmed via agarose gel electrophoresis. For purification, bacterial PCR products and amplicons >400 bp were processed with 0.6× Agencourt AMPure XP magnetic beads, while fungal PCR products and amplicons <400 bp were purified using 0.8× magnetic beads. Purified DNA was quantified using the Qubit 2.0 Fluorometer (Life Technologies, Carlsbad, CA, USA), mixed in a 1:1 ratio, and used for sequencing on the Illumina MiSeq 2 × 300 platform at a concentration of 20 pmol.

### 2.5. Analysis of Major Soil Properties

Soil organic carbon (SOC) was determined using the potassium dichromate oxidation method, total nitrogen (TN) using the Kjeldahl digestion method, available nitrogen (AN) using the alkaline hydrolysis diffusion method, and available phosphorus (AP) using extraction with 0.5 mol L^−1^ NaHCO_3_. Soil pH was measured using a pH meter. All analyses followed standard soil agrochemical procedures [23].

### 2.6. Statistical Analysis

Raw sequencing data were quality-checked using FastQC(version 0.11.5), merged using FLASH(version 1.2.3), and low-quality sequences were removed using Prinseq. Sequences with ≥97% similarity were clustered into operational taxonomic units (OTUs). One representative sequence was selected from each OTU and taxonomically assigned using the RDP Classifier (version 18).

All microbial community analyses were conducted in R (version 4.4.2). After rarefaction using the vegan package (version 2.6-10), α-diversity indices were calculated, and differences among treatments were assessed using one-way analysis of variance (ANOVA). Bray–Curtis dissimilarity matrices were used to evaluate β-diversity, which was visualized using principal coordinate analysis (PCoA). Differences in microbial community structure among treatments were tested using permutational multivariate analysis of variance (PERMANOVA) with 999 permutations.

Petal diagrams illustrating OTU distributions were generated using the plotrix package (version 3.8-13). Differential taxa from phylum to genus levels were identified using LEfSe analysis implemented in the microeco package (version 1.16.0), with an LDA threshold greater than 3.5. Putative functional annotation of bacterial and fungal communities was conducted using the FAPROTAX and FUNGuild databases, respectively. Spearman correlation and Mantel tests were applied to evaluate relationships between microbial communities and environmental variables. Co-occurrence network analysis based on Spearman correlations (|r| > 0.6, *p* < 0.05) was conducted using the psych package (version 2.6.3), and network visualization was performed using Gephi (version 0.9.7).

## 3. Results

### 3.1. Effects of Root Exudates from Different Plant Species on Microbial Diversity

**Figure 2:**
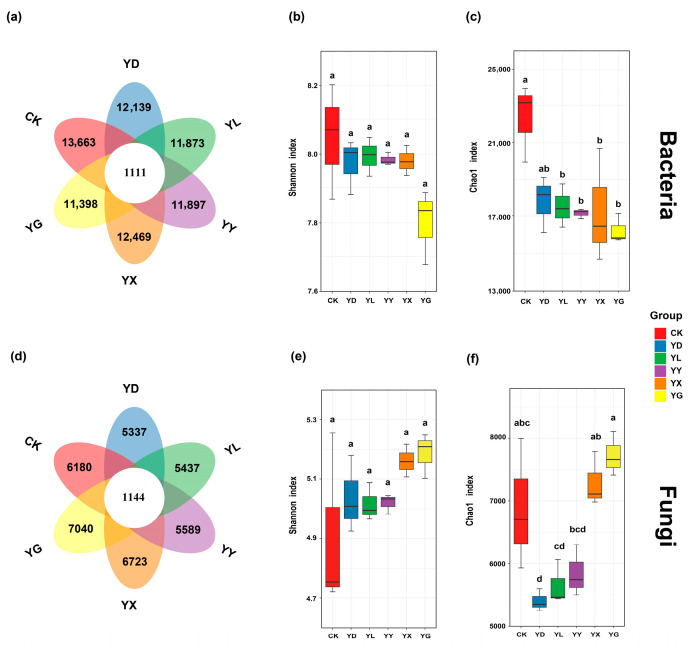
Petal diagrams show shared and unique OTUs of bacterial and fungal communities. Boxplot showing bacterial and fungal α-diversity (Chao 1 and Shannon indices) across different root exudate treatments. Subfigures: (**a**) bacterial OTUs, (**b**) bacterial Shannon Index, (**c**) bacterial Chao1 Index, (**d**) fungal OTUs, (**e**) fungal Shannon Index, (**f**) fungal Chao1 Index. Statistical differences were assessed using the Kruskal–Wallis test, followed by Dunn’s post hoc test with FDR correction. Different letters indicate significant differences (*p* < 0.05). CK: Control treatments; YD: *Stipa breviflora*; YL: *Artemisia frigida*; YY: *Cleistogenes songorica*; YX: *Convolvulus ammannii*; YG: *Heteropappus altaicus*.

In the control (CK) samples, a total of 13,363 bacterial OTUs and 6180 fungal OTUs were detected. Among the six treatments, 1111 bacterial OTUs and 1114 fungal OTUs were shared (Figure 2a,d). Compared with the control (CK), bacterial OTU richness decreased under all five root exudate treatments (Figure 2a). Fungal OTU richness declined under the *S. breviflora* (YD), *A. frigida* (YL), and *C. songorica* (YY) treatments, whereas it increased under the *C. ammannii* (YX) and *H. altaicus* (YG) treatments (Figure 2d). Shannon diversity indices of both bacterial and fungal communities did not differ significantly among treatments (Figure 2b,e). Root exudates from *S. breviflora* significantly reduced fungal richness (*p* < 0.05) but had no significant effect on bacterial richness. Root exudates from *A. frigida* and *C. songorica* significantly reduced both bacterial and fungal richness (*p* < 0.05). In contrast, root exudates from *C. ammannii* and *H. altaicus* significantly reduced bacterial richness but had no significant effect on fungal richness (Figure 2c,f).

**Figure 3:**
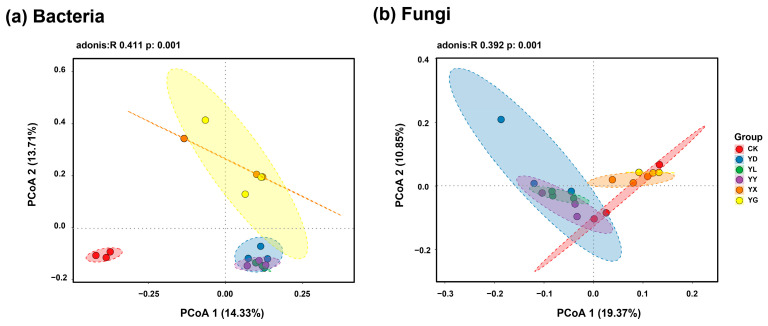
Principal Coordinate Analysis (PCoA) of bacterial (**a**) and fungal (**b**) communities based on Bray–Curtis dissimilarity. Each colored dot represents an individual sample from a specific root exudate treatment. CK: Control treatments; YD: *Stipa breviflora*; YL: *Artemisia frigida*; YY: *Cleistogenes songorica*; YX: *Convolvulus ammannii*; YG: *Heteropappus altaicus*.

Principal coordinate analysis (PCoA) based on Bray–Curtis distances revealed distinct clustering patterns of microbial communities among root exudate treatments (Figure 3). Bacterial communities exhibited greater compositional variation than fungal communities. For bacteria, samples from the YD, YL, and YY treatments clustered closely and were clearly separated from those of the YX and YG treatments, with all treatments distinct from the control (CK) (Figure 3a). For fungi, samples from CK, YD, YL, and YY clustered together, whereas YX and YG formed a separate group (Figure 3b).

### 3.2. Effects of Root Exudates from Different Plant Species on Microbial Community Composition and Species-Level Differences

**Figure 4:**
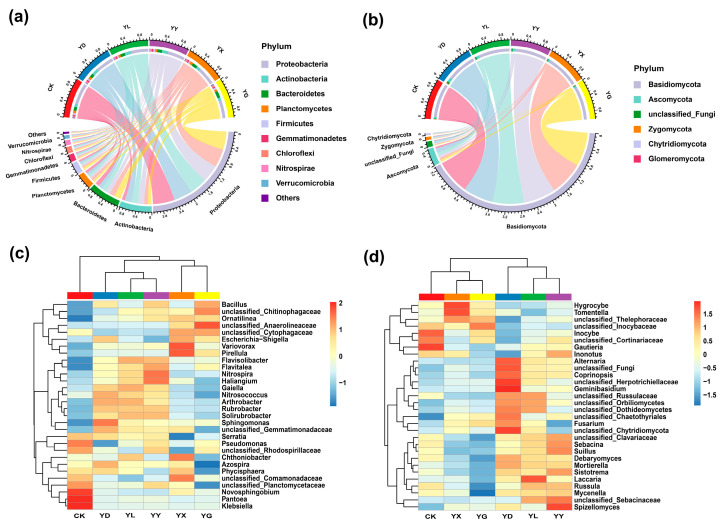
Relative abundance of dominant bacterial (**a**) and fungal (**b**) phyla. The chord diagram illustrates the phyla with relatively high abundance under different root exudate treatments. The clustering heatmap shows the top 30 genera under different treatment conditions for bacteria (**c**) and fungi (**d**). CK: Control treatments; YD: *Stipa breviflora*; YL: *Artemisia frigida*; YY: *Cleistogenes songorica*; YX: *Convolvulus ammannii*; YG: *Heteropappus altaicus*.

At the phylum level, bacterial communities in the control (CK) were dominated by Proteobacteria, Actinobacteria, Bacteroidetes, Planctomycetes, and Firmicutes (Figure 4a). Although the dominant bacterial phyla were consistent across treatments, the relative abundance of Proteobacteria decreased significantly under all root exudate treatments (*p* < 0.05), whereas Actinobacteria increased significantly in YD, YL, and YY (*p* < 0.05). Fungal communities in CK were mainly composed of Basidiomycota, Ascomycota, Zygomycota, and Chytridiomycota (Figure 4b). Root exudate treatments did not alter the dominant fungal phyla; however, the relative abundance of Basidiomycota decreased significantly in YD (*p* < 0.05), while Ascomycota and Zygomycota increased significantly in YD, YL, and YY (*p* < 0.05).

Hierarchical clustering based on the top 30 genera showed distinct grouping patterns among treatments (Figure 4c,d). For bacteria, YL and YY clustered together first, followed by YD, then YX and YG, with CK forming a separate cluster. For fungi, samples clustered into two major groups: one comprising YX, YG, and CK, and the other comprising YL, YY, and YD.

**Figure 5:**
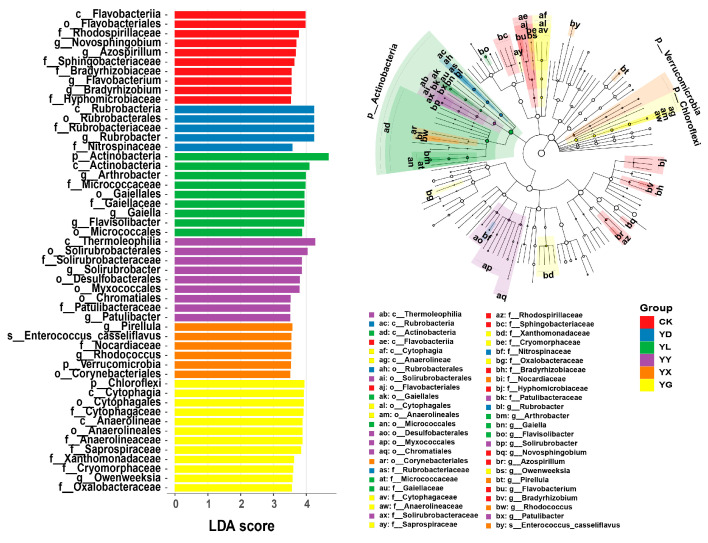
LEfSe analysis of bacterial indicator taxa under different root exudate treatments. Differentially abundant bacterial taxa identified by LEfSe with LDA scores > 3.5. CK: Control treatments; YD: *Stipa breviflora*; YL: *Artemisia frigida*; YY: *Cleistogenes songorica*; YX: *Convolvulus ammannii*; YG: *Heteropappus altaicus*.

LEfSe analysis identified 51 bacterial taxa with LDA scores > 3.5 (Figure 5). Of these taxa, 10 taxa were enriched in CK, while YD, YL, YY, YX, and YG enriched 5, 9, 9, 6, and 12 taxa, respectively. At the genus level, *Novosphingobium*, *Azospirillum*, *Flavobacterium*, and *Bradyrhizobium* were enriched in CK; *Rubrobacter* in YD; *Arthrobacter*, *Gaiella*, and *Flavisolibacter* in YL; *Solirubrobacter* and *Patulibacter* in YY; *Pirellula* and *Rhodococcus* in YX; and *Owenweeksia* in YG.

**Figure 6:**
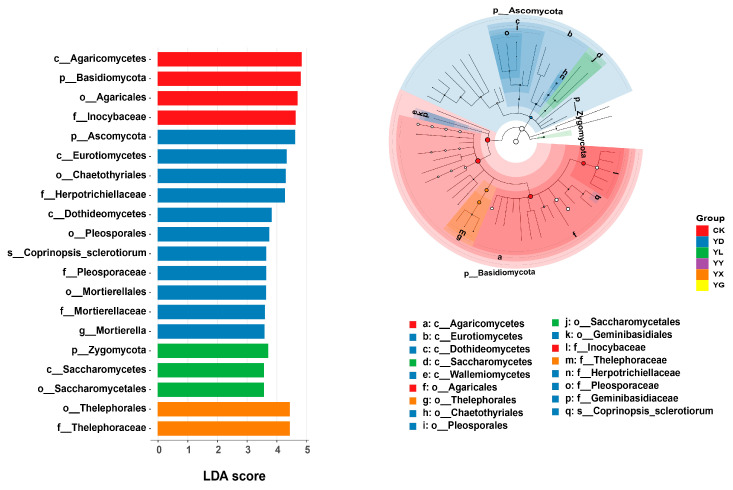
LEfSe analysis of fungal indicator taxa under different root exudate treatments. Differentially abundant fungal taxa identified by LEfSe with LDA scores > 3.5. CK: Control treatments; YD: *Stipa breviflora*; YL: *Artemisia frigida*; YY: *Cleistogenes songorica*; YX: *Convolvulus ammannii*; YG: *Heteropappus altaicus*.

For fungi, 20 taxa with LDA scores > 3.5 were identified (Figure 6). 4 taxa were enriched in CK, 11 in YD, 3 in YL, and 2 in YX, whereas no indicator taxa were detected in YY or YG. At the phylum level, Basidiomycota was enriched in CK, Ascomycota in YD, and Zygomycota in YL.

### 3.3. Predicted Microbial Functional Shifts Under Root Exudate Treatments

**Figure 7:**
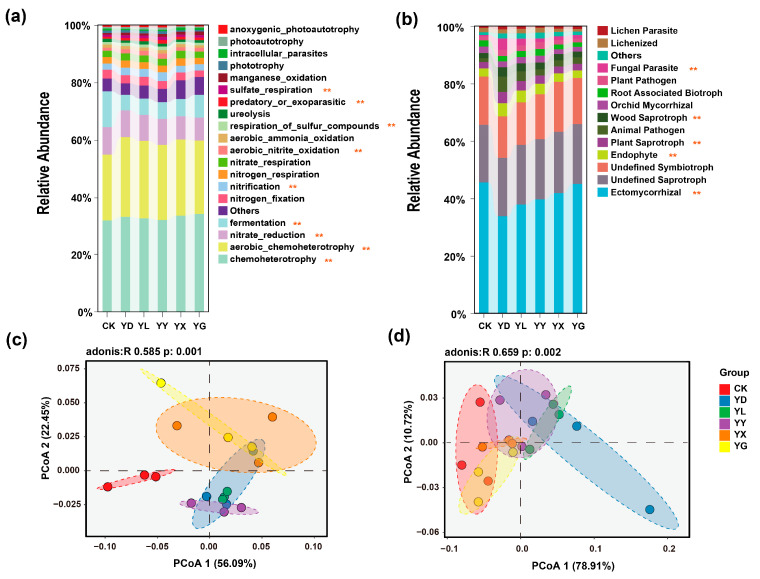
Predicted functional profiles and functional community patterns of bacterial and fungal communities under different root exudate treatments. (**a**) Relative abundance of bacterial functional groups predicted by FAPROTAX. (**b**) Relative abundance of fungal functional guilds predicted by FUNGuild. (**c**) Principal coordinate analysis (PCoA) of bacterial functional communities based on Bray–Curtis distances. (**d**) Principal coordinate analysis (PCoA) of fungal functional communities based on Bray–Curtis distances. Significant differences between groups are indicated by star symbols (**, *p* < 0.05).

FAPROTAX-based functional prediction suggested that, compared with the control (CK), the addition of root exudates was predicted to increase the relative abundance of bacterial functional groups associated with chemoheterotrophy, aerobic chemoheterotrophy, nitrification, aerobic nitrite oxidation, respiration of sulfur compounds, predatory or exoparasitic lifestyles, and sulfate respiration. In contrast, bacterial functions related to nitrate reduction and fermentation were predicted to decrease (Figure 7a).

FUNGuild analysis, which similarly predicts fungal functional guilds from taxonomic data, indicated that the relative abundances of endophytes, plant saprotrophs, wood saprotrophs, and fungal parasites were predicted to increase following root exudate addition, whereas ectomycorrhizal fungi were predicted to decrease (Figure 7b). Functional clustering patterns of bacterial communities remained generally similar among treatments, whereas the separation among fungal functional communities was predicted to be reduced (Figure 7c,d).

### 3.4. Microbial Co-Occurrence Networks Under Root Exudate Treatments

**Figure 8:**
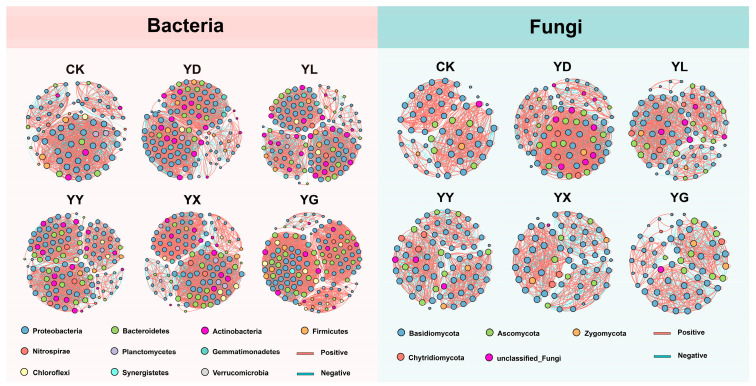
Co-occurrence networks of bacterial and fungal communities under different root exudate treatments. Networks were constructed based on OTUs with relative abundances > 0.2%. Node colors indicate phylum-level taxonomy, and edges represent significant correlations (positive or negative) between OTUs.

Co-occurrence networks were constructed using OTUs with relative abundance > 0.2% (Figure 8). Compared with CK, all root exudate treatments showed increased numbers of nodes, edges, and average degree in bacterial networks, indicating higher network complexity. The number of positive edges was highest in the YX and YG treatments. For fungal networks, YD, YL, and YY exhibited increased node numbers, edge numbers, and average degree relative to CK, whereas YX and YG showed reduced edge numbers and average degree, indicating lower network complexity (Table 1).

### 3.5. Effects of Root Exudates from Different Plant Species on Soil Properties

Root exudate treatments had no significant effects on soil organic carbon (SOC), total nitrogen (TN), or C/N ratio (Table 2). However, all five root exudate treatments significantly increased available nitrogen (AN) and significantly decreased soil pH (*p* < 0.05). The magnitude of pH reduction differed among treatments, with *H. altaicus* showing the strongest effect and *S. breviflora* the weakest. Effects on available phosphorus (AP) varied among species. Root exudates from *C. songorica* and *C. ammannii* significantly increased AP, whereas those from *S. breviflora*, *A. frigida*, and *H. altaicus* showed non-significant increases.

### 3.6. Relationships Between Microbial Communities and Soil Properties

**Figure 9:**
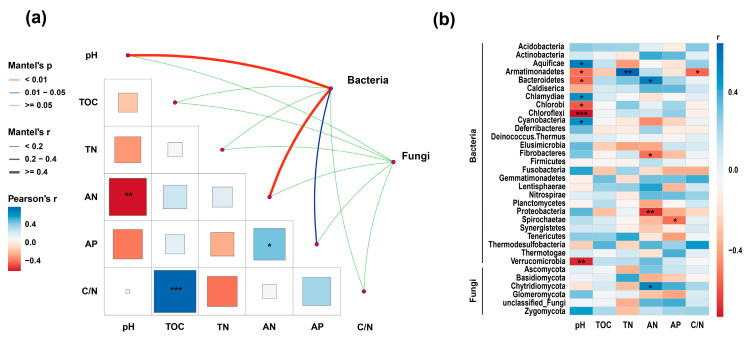
Mantel test and Spearman correlation analysis linking soil properties with bacterial and fungal communities. (**a**) Mantel test between soil variables and microbial community composition. (**b**) Spearman correlations between dominant microbial phyla and soil properties. Significant differences between groups are indicated by star symbols (*, *p* < 0.05; **, *p* < 0.01; ***, *p* < 0.001).

Mantel tests indicated that soil pH and AN were strongly associated with bacterial community composition (*p* < 0.01), while AP showed a weaker but significant association (*p* < 0.05). No soil variables were significantly correlated with fungal community composition (Figure 9a).

At the phylum level, Spearman correlation analysis revealed that Aquificae, Chlamydiae, and Cyanobacteria were positively correlated with soil pH, whereas Armatimonadetes, Bacteroidetes, Chlorobi, Chloroflexi, and Verrucomicrobia were negatively correlated with pH (Figure 9b). Armatimonadetes showed a positive correlation with TN and a negative correlation with C/N ratio. Bacteroidetes was positively correlated with AN, whereas Fibrobacteres and Proteobacteria were negatively correlated with AN. Spirochaetae was negatively correlated with AP. Among fungi, Chytridiomycota showed a significant positive correlation with AN.

## 4. Discussion

### 4.1. Effects of Root Exudates on Rhizosphere Microbial Communities

The rhizosphere is the core interface where plants, soil, and microorganisms interact intensively. Due to the influence of root activity, rhizosphere soils often differ markedly from bulk soils in physicochemical properties and microbial community structure [24]. Numerous studies have reported that microbial diversity and richness are generally higher in bulk soils than in rhizosphere soils, as demonstrated for mangrove species such as *Kandelia obovata*, *Avicennia marina*, and *Aegiceras corniculatum* [25], as well as for *Phyllostachys edulis* [26], *Rhododendron moulmainense* [27], and *Paris polyphylla* [28]. Soil conditions, plant species, and plant genotypes have all been shown to differentially influence the structure and activity of rhizosphere microbial communities [29,30]. Among these factors, root exudates are widely recognized as a major driver underlying the divergence between rhizosphere and bulk soil microbial communities [31,32]. In the present study, soils treated with root exudates collected from the five plant species showed significant shifts in microbial community composition compared with the control, along with a decrease in microbial diversity and richness, particularly in bacterial communities. These findings further demonstrate that root exudates are a key factor driving the differentiation between rhizosphere and bulk soil microbial communities.

PCoA and hierarchical clustering analyses indicated that, among the five plant species, root exudates from the dominant species *A. frigida* and *C. songorica* exerted the most similar effects on both bacterial and fungal communities, and these effects were also comparable to those induced by the constructive species *S. breviflora*. In contrast, root exudates from the accompanying species *C. ammannii* and *H. altaicus* showed higher similarity to each other but differed clearly from those of the dominant species.

In bulk desert steppe soils without root exudate addition, indicator bacterial taxa were mainly affiliated with Proteobacteria, including *Novosphingobium*, *Azospirillum*, and *Bradyrhizobium*, as well as *Flavobacterium* belonging to Bacteroidetes. Following root exudate addition, soils treated with exudates from the three dominant species exhibited a significant increase in the relative abundance of several Actinobacteria genera. Specifically, *Rubrobacter* was predominantly enriched in soils treated with exudates from *S. breviflora*, *Arthrobacter*, *Gaiella*, and *Flavisolibacter* in those treated with *A. frigida*, and *Solirubrobacter* and *Patulibacter* in soils treated with *C. songorica*. In contrast, root exudates from the two accompanying species did not lead to a marked increase in overall Actinobacteria abundance, but were instead characterized by the enrichment of *Pirellula* in *C. ammannii* and *Owenweeksia* in *H. altaicus*. Differences in both the quantity and chemical composition of root exudates among plant species [33,34], together with microbial substrate preferences [35], likely account for these divergent microbial responses [36].

Actinobacteria are known to play important roles in organic matter decomposition, nitrogen fixation, antibiotic and phytohormone production, pathogen suppression, and soil structure improvement [37,38,39,40]. Accordingly, the enrichment of Actinobacteria induced by root exudates from dominant species may be associated with the maintenance of their dominance in desert steppe communities.

### 4.2. Effects of Root Exudates on the Rhizosphere Microenvironment

Beyond their influence on microbial community composition, root exudates play an important role in regulating rhizosphere microenvironments, including nutrient mobilization and soil structure formation [41,42,43]. In this study, root exudates from all five plant species significantly reduced soil pH and increased the contents of available nitrogen (AN) and available phosphorus (AP), indicating that these plants can alleviate nitrogen and phosphorus limitation and alkaline stress in desert steppe soils through root exudation. Yinghan et al. [44] demonstrated that, as a result of root exudation, rhizosphere soils of three halophytic plant species contained higher concentrations of total carbon (TC), total nitrogen (TN), and available phosphorus (AP) than bulk soils. Organic acids released in root exudates can decrease soil pH through proton release [45], which is consistent with the patterns observed in the present study. These findings indicate that, although plants cannot relocate to more favorable habitats as animals do, they can enhance their tolerance to adverse environmental conditions by modifying the rhizosphere microenvironment via root exudation.

Correlation analyses further revealed that Proteobacteria abundance was positively correlated with soil pH but negatively correlated with total organic carbon, AN, and AP, whereas Actinobacteria abundance showed positive correlations with AN and AP. Tang et al. [46] suggested that plants can attract Actinobacteria by supplying energy-rich root exudates. In the rhizosphere, *Gaiella* may co-occur with nitrogen-fixing bacteria such as *Bacillus*, potentially enhancing nitrogen acquisition [47]. *Arthrobacter* preferentially utilizes nitrate and regulates nitrogen assimilation through the glutamine synthetase gene (glnA), thereby improving nitrogen use efficiency [48]. Moreover, *Arthrobacter* strains isolated from ginseng rhizospheres have been shown to produce indole-3-acetic acid and solubilize phosphorus and potassium [49]. *Rubrobacter* and *Solirubrobacter* have been reported to exhibit potential roles in enhancing root-associated nitrogen accumulation in *Stipa grandis* and *Cleistogenes squarrosa* rhizospheres [50].

Collectively, these results indicate that plants can selectively recruit beneficial microorganisms through root exudation to mitigate adverse environmental conditions [51,52,53,54]. Combined with FAPROTAX and FUNGuild functional predictions, the observed functional shifts suggest that root exudates drive the selective assembly of microbial functional groups that promote nutrient transformations beneficial to plant growth (e.g., nitrification and sulfur respiration), while suppressing pathways associated with nutrient loss (e.g., nitrate reduction).

### 4.3. The Ecological Role of Plants in a Community Goes Beyond Their Phylogenetic Role in Shaping Microbial Responses to Root Exudates

Root exudates from the three dominant species exerted similar effects on microbial functions, whereas the two accompanying species also showed similar but distinct functional impacts compared with dominant species. This pattern may reflect the release of similar exudate compounds (e.g., tributyl acetylcitrate, erucamide, and 1,2-dimethylbenzene) by *S. breviflora*, *A. frigida*, and *C. songorica*, which collectively shape core microbial functions [55]. Notably, although *S. breviflora* and *C. songorica* belong to Poaceae and *A. frigida* and *H. altaicus* belong to Asteraceae, the effects of root exudates on microbial communities were more strongly associated with ecological roles (dominant vs. accompanying species) than with phylogenetic relatedness. This contrasts with findings by G. et al. [56], who reported similar rhizosphere microbial functions among species within the same family, and with Yang et al. [57], who observed higher microbial similarity among grasses than legumes. Such discrepancies may arise because the plant species examined here coexist within the same community and share long-term interactions.

Co-occurrence network analysis showed that root exudates from all five species increased bacterial network complexity, as indicated by higher node numbers, edge numbers, and average degree. This suggests that root exudates promote bacterial recruitment and intensify microbial interactions in the rhizosphere. Compared with dominant species, accompanying species exhibited a higher proportion of positive correlations, indicating the formation of more cooperative microbial networks, which may represent an adaptive survival strategy. In contrast, root exudates from the dominant species increased both bacterial and fungal network complexity, whereas those from accompanying species reduced fungal network complexity. These results suggest that dominant species in the Inner Mongolian desert steppe tend to enhance competitive ability by directly stimulating specific functional microbial groups (e.g., nitrifiers and sulfur-respiring bacteria) to improve resource acquisition efficiency. In contrast, accompanying species may favor the establishment of stable, cooperative microbial communities to enhance resistance to environmental stress, such as nutrient limitation. Such divergent root exudate-microbe interaction strategies likely contribute to species coexistence and the maintenance of ecosystem functioning in desert steppe communities.

### 4.4. Limitations and Future Research Directions

This study investigated the effects of whole root exudates from different plant species on soil microbial communities and nutrient dynamics by collecting exudates using a hydroponic system, concentrating them, and applying them to intact soil. Several limitations should be considered. First, although hydroponic methods are commonly used for exudate collection, they cannot fully reproduce rhizosphere conditions in the field. Therefore, the observed microbial responses should be interpreted as potential effects of root exudates rather than exact representations of in situ processes. Second, functional predictions based on FAPROTAX and FUNGuild rely on taxonomic annotation and do not reflect actual gene expression, and thus represent potential rather than confirmed functions. Future studies should validate these findings under more realistic conditions, such as rhizobox experiments or in situ exudate collection [58,59]. The integration of metatranscriptomics or stable isotope probing would further enable direct assessment of microbial functional activity and strengthen the functional interpretation of root exudate effects.

## 5. Conclusions

Root exudates are a key driver of differences in microbial community composition and soil microenvironment between rhizosphere and bulk soils. In this study, root exudates from five desert steppe plant species had no significant effects on bacterial or fungal diversity, but significantly increased bacterial richness and markedly altered the composition of both bacterial and fungal communities. Overall, root exudates from the three dominant species exerted broadly similar effects on soil microbial communities, consistently increasing the relative abundance of Actinobacteria, albeit with distinct genus-level enrichment patterns. Specifically, exudates from *S. breviflora* primarily enriched *Rubrobacter*, those from *A. frigida* favored *Arthrobacter*, *Gaiella*, and *Flavisolibacter*, and those from *C. songorica* selectively enriched *Solirubrobacter* and *Patulibacter*. In contrast, the two accompanying species showed comparable effects on microbial communities but did not significantly increase Actinobacteria abundance, instead enriching *Pirellula* (Planctomycetota) in *C. ammannii*-treated soils and *Owenweeksia* (Proteobacteria) in *H. altaicus*–treated soils.

Across all species, root exudate addition significantly decreased soil pH and increased available nitrogen and phosphorus, identifying these variables as primary environmental factors shaping soil bacterial community composition. Collectively, these findings demonstrate that coexisting plant species within the same community can differentially modify soil microbial functional assemblages through root exudation of labile carbon substrates and signaling compounds. This process restructures microbial community composition, metabolic potential, and interaction networks, thereby regulating key biogeochemical cycles of carbon, nitrogen, sulfur, and other elements in the rhizosphere and ultimately influencing soil ecological functions and plant niche differentiation.

## Figures and Tables

**Table 1:** Topological properties of co-occurrence networks of soil bacterial and fungal communities.

Microbe	Sample	Node Num	Edge Num	Positive Edges Percent	Average Degree	Density	Clustering Coefficient
Bacteria	CK	82	1004	54.07	25.488	0.315	0.990
	YD	114	2023	52.35	35.491	0.314	0.993
	YL	124	1876	54.26	30.258	0.246	0.992
	YY	122	1851	52.84	30.344	0.251	0.992
	YX	117	2144	61.80	36.650	0.316	0.994
	YG	131	2674	70.68	40.824	0.314	0.995
Fungi	CK	58	589	71.14	20.310	0.356	0.982
	YD	81	1314	58.30	32.444	0.406	0.991
	YL	77	931	53.17	24.182	0.318	0.988
	YY	72	863	53.07	23.972	0.338	0.985
	YX	60	566	59.54	18.867	0.320	0.982
	YG	61	535	57.38	17.541	0.292	0.981

Note: CK: Control treatments; YD: Stipa breviflora; YL: Artemisia frigida; YY: Cleistogenes songorica; YX: Convolvulus ammannii; YG: Heteropappus altaicus.

**Table 2:** Main soil nutrients under different treatments.

Sample	SOC (g/kg)	TN (g/kg)	AN (mg/kg)	AP (mg/kg)	pH	C/N
CK	17.79 ± 0.06 ab	1.24 ± 0.03 a	31.83 ± 1.46 b	4.26 ± 0.99 b	7.81 ± 0.02 a	14.37 ± 0.35 ab
YD	14.64 ± 3.27 b	1.21 ± 0.07 a	42.53 ± 4.34 a	5.44 ± 1.07 ab	7.56 ± 0.02 b	11.99 ± 2.02 ab
YL	19.77 ± 0.21 a	1.32 ± 0.16 a	48.22 ± 0.94 a	5.64 ± 0.69 ab	7.49 ± 0.01 c	15.10 ± 1.90 ab
YY	16.33 ± 4.09 ab	1.11 ± 0.12 a	44.11 ± 3.49 a	6.77 ± 0.67 a	7.52 ± 0.02 c	15.01 ± 4.85 ab
YX	19.94 ± 3.22 a	1.29 ± 0.18 a	48.34 ± 2.34 a	6.47 ± 0.29 a	7.20 ± 0.01 e	15.47 ± 1.41 a
YG	13.70 ± 1.55 b	1.31 ± 0.05 a	44.52 ± 5.87 a	5.31 ± 0.90 ab	7.24 ± 0.02 d	10.48 ± 1.56 b

Note: Values are presented as mean ± standard error (*n* = 3). Different letters indicated statistically significant differences among treatments (*p* < 0.05, ANOVA followed by Duncan’s multiple range test). CK: Control treatments; YD: *Stipa breviflora*; YL: *Artemisia frigida*; YY: *Cleistogenes songorica*; YX: *Convolvulus ammannii*; YG: *Heteropappus altaicus*. SOC: soil organic carbon; TN: total nitrogen; AN: available nitrogen; AP: available phosphorus.

## Data Availability

The original contributions presented in this study are included in the article. Further inquiries can be directed to the corresponding author.
