# Correction: E et al. Root Exudates from Coexisting Plant Species Differentially Shape Soil Microbial Communities and Nutrient Dynamics in a Desert Steppe. *Microorganisms* 2026, *14*, 950

**DOI:** 10.3390/microorganisms14081634

**Published:** 2026-07-27

**Authors:** Leqing E, Guodong Han, Jie Liu, Xuefeng Gao

**Affiliations:** 1 College of Life Science and Technology, Inner Mongolia Normal University, Hohhot 010022, China; elqin0117@163.com (L.E.); lj18175071216@163.com (J.L.); 2 Key Laboratory of Biodiversity Conservation and Sustainable Utilization in Mongolian Plateau for College and University of Inner Mongolia Autonomous Region, Hohhot 010022, China; 3 College of Grassland Science, Inner Mongolia Agricultural University, Hohhot 010022, China; nmghanguodong@163.com

In the original publication [1], there was an error regarding the affiliations for Leqing E, Jie Liu and Xuefeng Gao. In addition to affiliation 1, the updated affiliations should include: Key Laboratory of Biodiversity Conservation and Sustainable Utilization in Mongolian Plateau for College and University of Inner Mongolia Autonomous Region, Hohhot 010022, China.

The authors state that the scientific conclusions are unaffected. This correction was approved by the Academic Editor. The original publication has also been updated.

## References

[1] E L., Han G., Liu J., Gao X. (2026). Root Exudates from Coexisting Plant Species Differentially Shape Soil Microbial Communities and Nutrient Dynamics in a Desert Steppe. Microorganisms.

